# MEMS vibrometer: Dynamic modeling of multimodal inertial transducers

**DOI:** 10.1038/s41598-025-90976-3

**Published:** 2025-02-26

**Authors:** Jan Niklas Haus, Zhengchun Zhu, Thomas Roloff, Liv Rittmeier, Sarah Bornemann, Michael Sinapius, Andreas Dietzel

**Affiliations:** 1 Institute of Microtechnology, Technische Universität Braunschweig, Braunschweig, Germany; 2 Institute of Mechanics and Adaptronics, Technische Universiät Braunschweig, Braunschweig, Germany; 3 Institute for Microsensors, Actuators and Systems, Universität Bremen, Bremen, Germany

**Keywords:** Electrical and electronic engineering, Characterization and analytical techniques

## Abstract

Guided ultrasonic wave-based structural health monitoring utilizes propagating elastic waves to identify, locate, and characterize damage within aviation structures. Fiber metal laminates, which are composite materials made by layering metal sheets with fiber-reinforced polymers, combine the high strength of composites with the ductility and impact resistance of metals. However, structural health monitoring methods suitable for these materials have to be developed, allowing to monitor also the inner laminate layers. Therefore, laminate-embedded MEMS vibrometers have been introduced recently. Due to the quasi-free operation of these inertial sensors, they are directly sensitive to the displacement induced by propagating guided ultrasonic waves. However, the multimodal excitation of the sensor’s core resonator, when exposed to ultrasound bursts, leads to a pseudo-nonlinear sensor response, which is attributed to the spectrum of guided ultrasonic waves and their interference with higher harmonics of the continuum resonator. The transfer behavior of the sensor can be improved by implementing electrical mode suppression. This research involves analytically modeling the continuous resonator with multiple aggregated resonators, numerically simulating sensor responses to 100 kHz ultrasound bursts, and using a laser scanning micro vibrometer setup for experimental validation, providing a deeper understanding of MEMS vibrometer dynamics for ultrasonic monitoring and demonstrating their applicability.

## Introduction

**Fig. 1:**
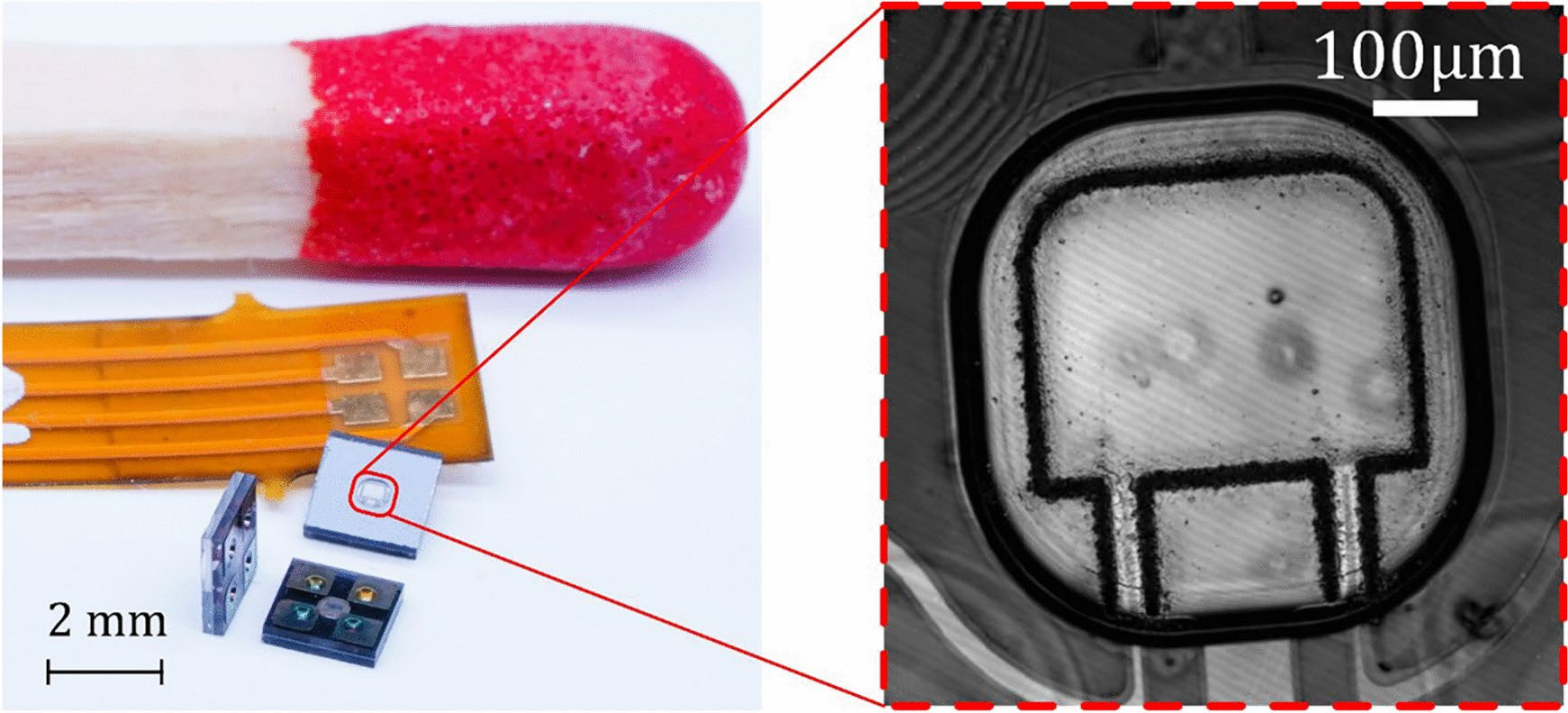
left: MEMS vibrometers in different poses for soldering to flexible PCB substrate. Right: Micrograph of the silicon cantilever resonator.

Structural health monitoring (SHM) using guided ultrasonic waves (GUW) is an established non-destructive testing technique for plates and plate-like structures^1–5^. However, SHM for fiber-metal laminates (FML) is still the subject of current research^6^. In particular, it requires structurally integrated sensors to monitor also the inner FML layers^7,8^. While piezoelectric wafer active sensors (PWAS) can resolve surface waves even beyond the 100 kHz range^9^, their ability to be integrated into FML is limited by mismatch of acoustic impedances and typical dimensions of these transducers^10,11^. Propagating GUW occur as symmetric, anti-symmetric and shear horizontal wave modes, referred to as *S-mode*, *A-mode* and *SH-mode* waves. The multitude of occurring wave modes increases the signal complexity. The ability to distinguish between these modes is key to more distinct GUW based SHM. Utilizing optical vibrometers^12^, the structure’s GUW-induced motion which can consist of superimposed modes can be decomposed into in-plane and out-of-plane motion^13^*.* Inertial MEMS sensors, which are a popular choice for SHM of civil structures as e. g. accelerometers for bridges^14^ offer directional selectivity. However, the bandwidth of MEMS accelerometers is typically limited to few kHz range and they are therefore not applicable for SHM using GUW. However, with a novel sensor class referred to as “MEMS vibrometers” (see Fig. 1), high-frequency displacements, as introduced by structure propagating GUW could be recorded in quasi-free-excitation^15^. Structure-integrable MEMS vibrometers have been developed, which could resolve GUW bursts with a center frequency of 100 kHz. Due to their small size of 2.2 mm × 2.2 mm × 410 µm, the MEMS vibrometers can be integrated into the layup of FML plates without influencing the linear wave propagation of GUW^16^. With good acoustic impedance matching of the MEMS sensor to the surrounding structure also non-linear sensor-GUW interaction is reduced^17^. However, in these previous studies the inherent structural dynamic behavior of the MEMS vibrometer’s continuous core resonator have not yet been investigated.

## Theoretical background

### The superposition model for multi-modal resonators

Traditionally, MEMS inertial sensors are either accelerometers or gyroscopes but seismometers can complement this class. Recently, a MEMS gyroscope has been studied using a bulk-acoustic resonator in the MHz range^18^. Also, a MEMS accelerometer for gravity and seismic measurements using a high-frequency vibrating beam with very high sensitivity to acceleration at low frequencies has been reported^19^. The well-known and widely established linear inertial accelerometer only operates well below the lowest natural frequency of its micromechanical core, where the sensor output has a near-linear relationship to the acceleration acting on the sensor base. With the low-pass characteristics of acceleration sensing, the bandwidth is limited to a frequency range also called the quasi-static regime, in which the input–output phase shift remains close to 0°.

The MEMS vibrometer however operates above the first resonance frequency of the micromechanical core, where the relative displacement of the spring-loaded proof mass, defining the output signal, has a linear relation to the displacement of its base, defining the input signal. In this case the sensor has a high-pass characteristic and the bandwidth is limited by its lower cut off frequency. The corresponding frequency range is referred to as the quasi-free regime, in which the input–output phase shift is close to -180°. A spring-mass-damper system with a single degree of freedom (1-DOF) can be described by the differential equation

1 $$m\cdot \frac{{d}^{2}{x}_{r}\left(t\right)}{d{t}^{2}}+b\cdot \frac{d{x}_{r}\left(t\right)}{dt}+k\cdot {x}_{r}\left(t\right)= -m\cdot \frac{{d}^{2}{x}_{f}\left(t\right)}{d{t}^{2}}$$

where *x*_*f*_ represents the foot point displacement and *x*_*r*_ is the relative displacement between the proof mass and the base. The transfer function^15^ for a displacement stimulus is given as

2 $${G}_{displ}(i\omega ) = \frac{-{(i\omega )}^{2}\cdot m}{-{\omega }^{2}\cdot m+i\omega \cdot b+k}$$

**Fig. 2:**
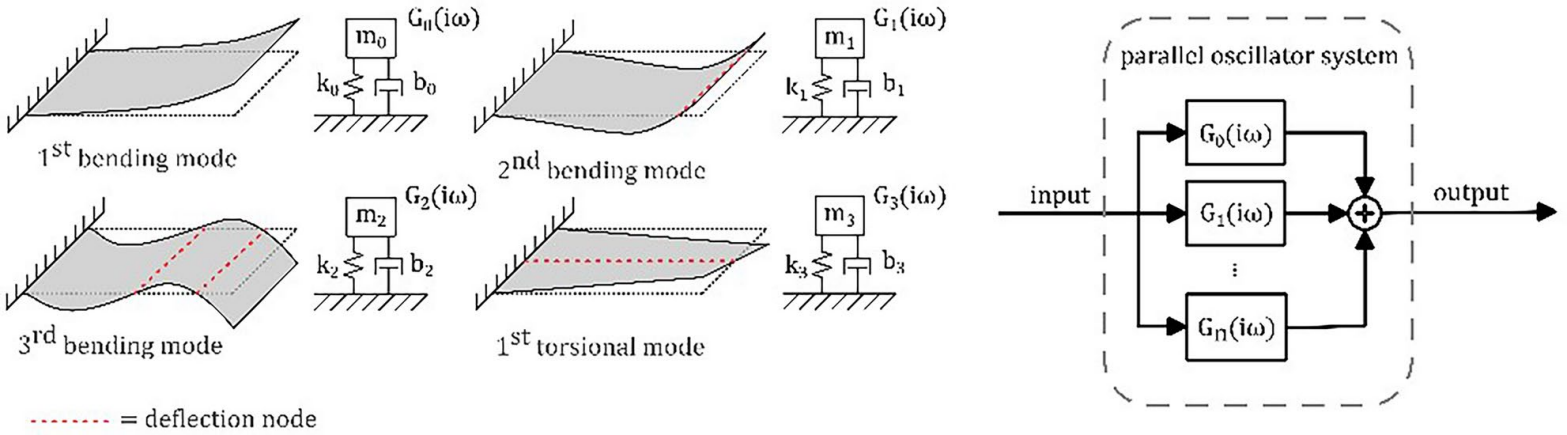
Dynamic model of a multimodal resonator (2D cantilever). Individual mode shapes are represented by individual spring-mass-damper models and their transfer functions which add up to the system’s transfer function.

**Fig. 3:**
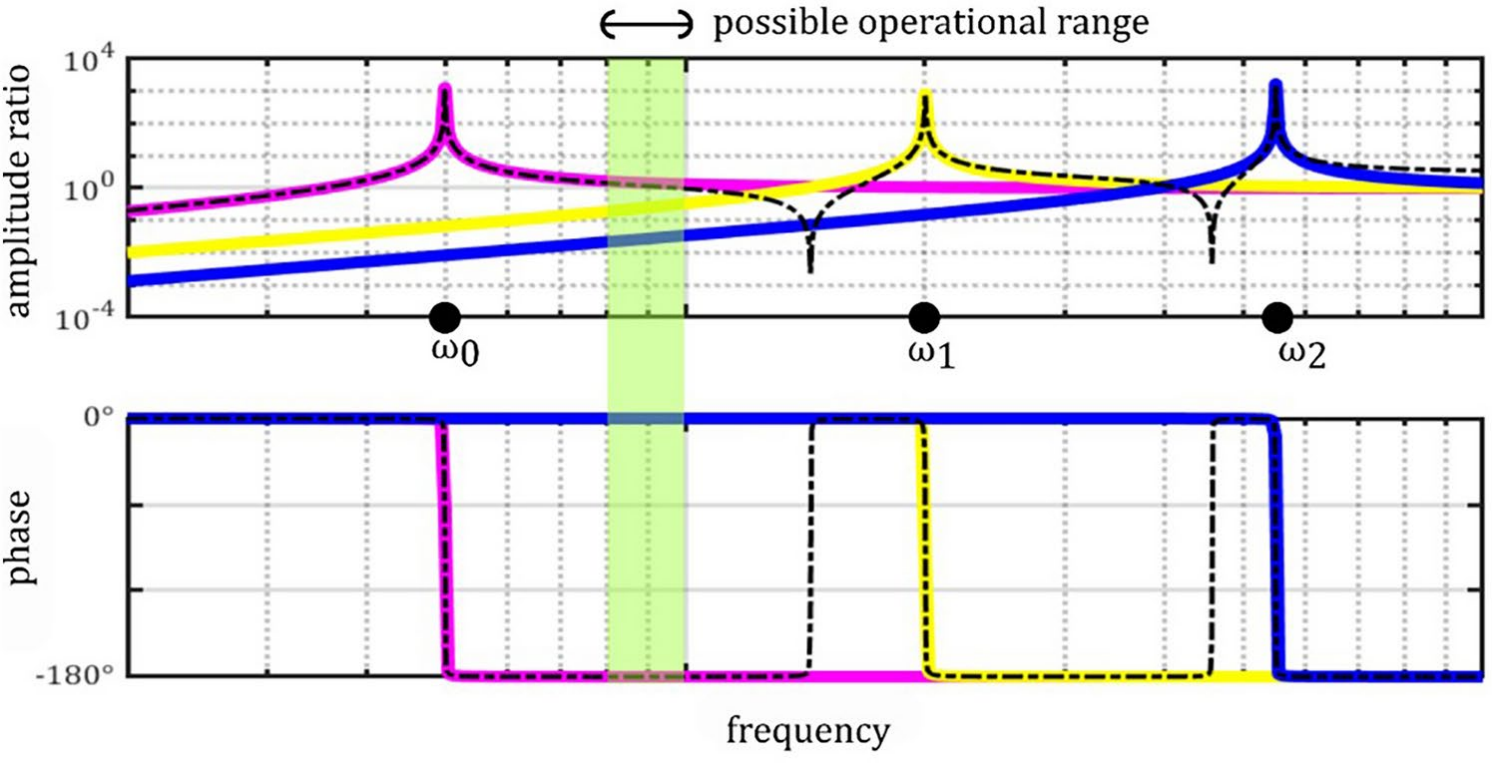
Analytically calculated transfer behavior of a generic multimodal resonator with amplitude and phase spectra in the vicinity of the first three resonances, assuming identical amplitudes in all resonances. 1^st^ mode resonator (pink), 2^nd^ mode resonator (yellow), 3^rd^ mode resonator (blue), sum (black). The traces are calculated from Eq. 2, assuming ${\omega }_{i}=\sqrt{{k}_{i}/{m}_{i}}$. The respective angular frequencies are ${\omega }_{0}=5\,\text{rad/s},\ {\omega }_{1}=20\,\text{rad/s},\ {\omega }_{2}=55\, \text{rad/s}$ for $i= 0, 1, 2$. The damping coefficient $b=0.01$.

In contrast to the model of a 1-DOF spring-mass-damper system, a real MEMS oscillator structure is continuous and thus has infinite resonance modes^20^. This can be modeled as multiple superimposed 1-DOF spring-mass-damper systems with one resonant frequency each, as illustrated in Fig. 2 for cantilevers. The transfer function of a parallel oscillator system is given as $\sum_{i=0}^{\infty }{G}_{i}(i\omega )$. The general course of amplitude and phase spectra of such a system is given in Fig. 3. A possible operational frequency range for receiving signals proportional to displacements is indicated above the first resonance frequency, outside the vicinity of all (anti-) resonances, and predominantly constant course of amplitude and phase.

### Ultrasound sine burst excitation

For SHM ultrasound in form of narrow-band sine bursts are injected into the tested structure. The resulting structure-propagating waves are referred to as GUW. A windowed sine burst *In(t)* with a center frequency of ${\omega }^{*}$ can be described as a multiplication of a sine function $f(t) = A \cdot sin({\omega }^{*}t)$ with a Hanning window function $hw(t)=0.5 (1 - cos(\omega \cdot t))$ where $2\pi /\omega$ represents the window length.

3 $$In\left(t\right)=f\left(t\right)\cdot hw\left(t\right)=\frac{A}{4}\left\{\begin{aligned}(2sin({\omega }^{*}t)-sin(({\omega }^{*}-\omega )t)-sin(({\omega }^{*}+\omega )t)&), \quad 0\le t\le \frac{2\pi }{\omega }\\ &0, \quad t<0;t>\frac{2\pi }{\omega }\end{aligned}\right.$$

The 5-cyclic GUW burst used in this article has a center frequency of ${\omega }^{*}/2\pi =100\,\text{kHz}$ and a window length of 50 µs resulting from $\omega ={\omega }^{*}/5$.

### Vibrometer as transducer for GUW bursts

When a multi-modal resonator, as described in Sect. “The superposition model for multi-modal resonators” is excited by a GUW burst as described in Sect.  “Ultrasound sine burst excitation”, each resonator mode can be excited, if the excitation has corresponding frequency contributions^21^. The vibrometer’s transfer function *G(iω)* should ideally remain constant in the excitation spectrum to ensure a linear and non-dispersive response. Exemplarily for a resonator which is suitable as a vibrometer for GUW with a center frequency of ${\omega }^{*}$, a generic oscillator system with a first natural frequency of $0.1{\omega }^{*}$ and a second natural frequency of $10{\omega }^{*}$ is modelled according to Sect.  “The superposition model for multi-modal resonators”. Higher resonator modes are neglected due to their near-zero transfer functions at ${\omega }^{*}$.

**Fig. 4:**
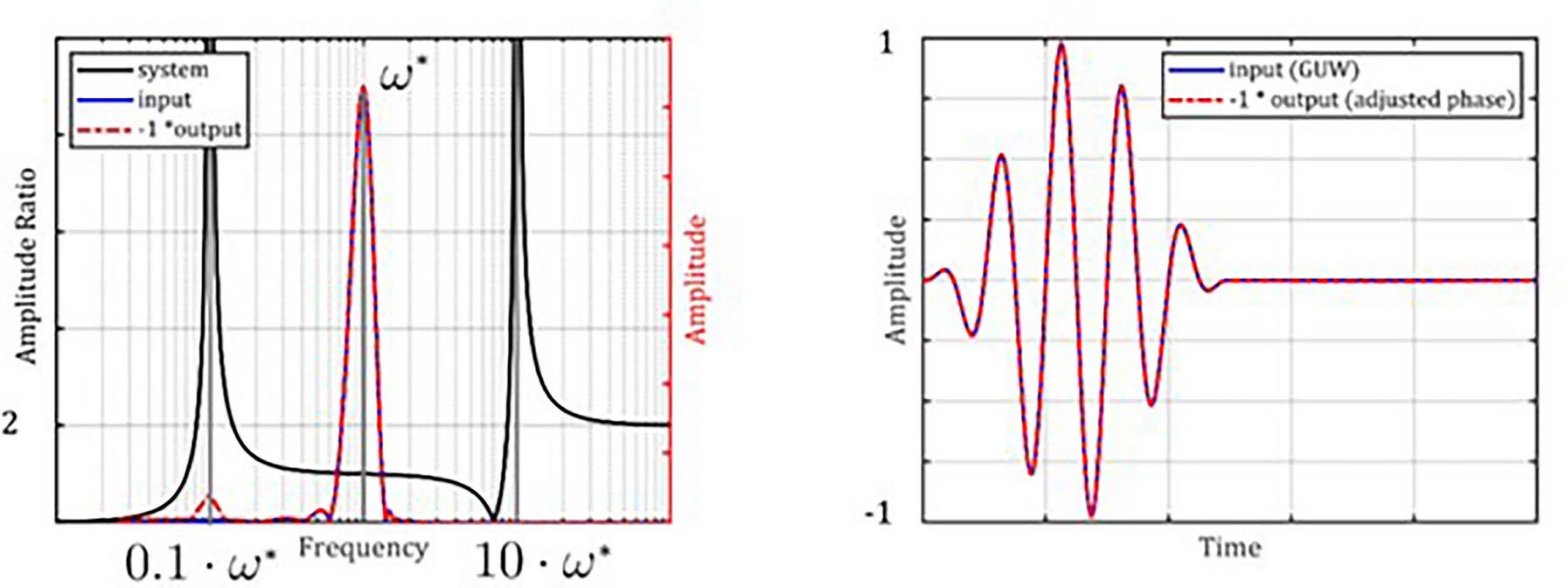
Transfer behavior of a generic multimodal oscillator with resonances at $0.1{ \omega }^{*}$ and $10 {\omega }^{*}$ exposed to a GUW-like burst with a center frequency of ${\omega }^{*}$. The output contains a small contribution from the first resonance.

The calculated case, presented in Fig. 4 shows how the burst input leads to a burst output signal. Input and output almost perfectly correlate when ${\omega }^{*}$ has sufficient distance to the resonator’s natural frequencies. For better visual comparability, the output signal is shifted by 180°.

## Materials and methods

### MEMS vibrometer design and fabrication

**Fig. 5:**
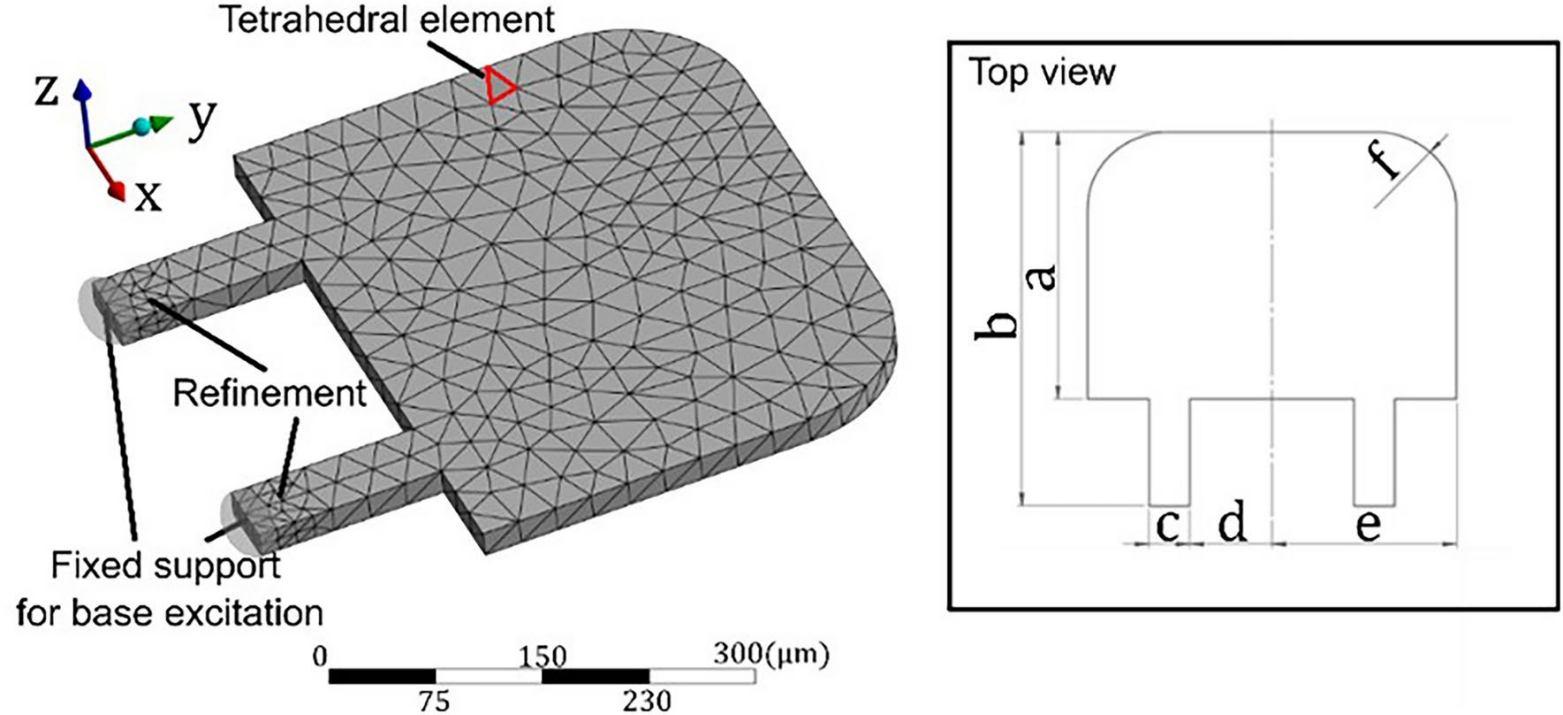
Representative 3D model of the core resonator in SOLIDWORKS with all necessary measures (a = 490 µm, b = 350 µm, c = 50 µm, d = 100 µm, e = 225 µm, f = 100 µm, thickness = 20 µm).

The resonator was designed as a cantilever with a two slender rod suspension carrying piezoresistive paths to provide an almost constant sensitivity in the frequency band of the GUW. In comparison with an earlier design^15^ the mechanical stresses are concentrated in the rods. Moreover, in the case of torsional deformation the stress values within the rods will have opposite signs and cancel out in the Wheatstone circuit readout. Thus, the first torsional resonance mode will not contribute to the sensor signal. The effect of this “mode rejection” on the senor’s dynamic response is discussed in Section “Discussion”. The cantilever was dimensioned as sketched in Figure 5 based on finite element based modal analysis as will be discussed in Section “Modal Analysis and Electrical Mode Rejection”.

**Fig. 6:**
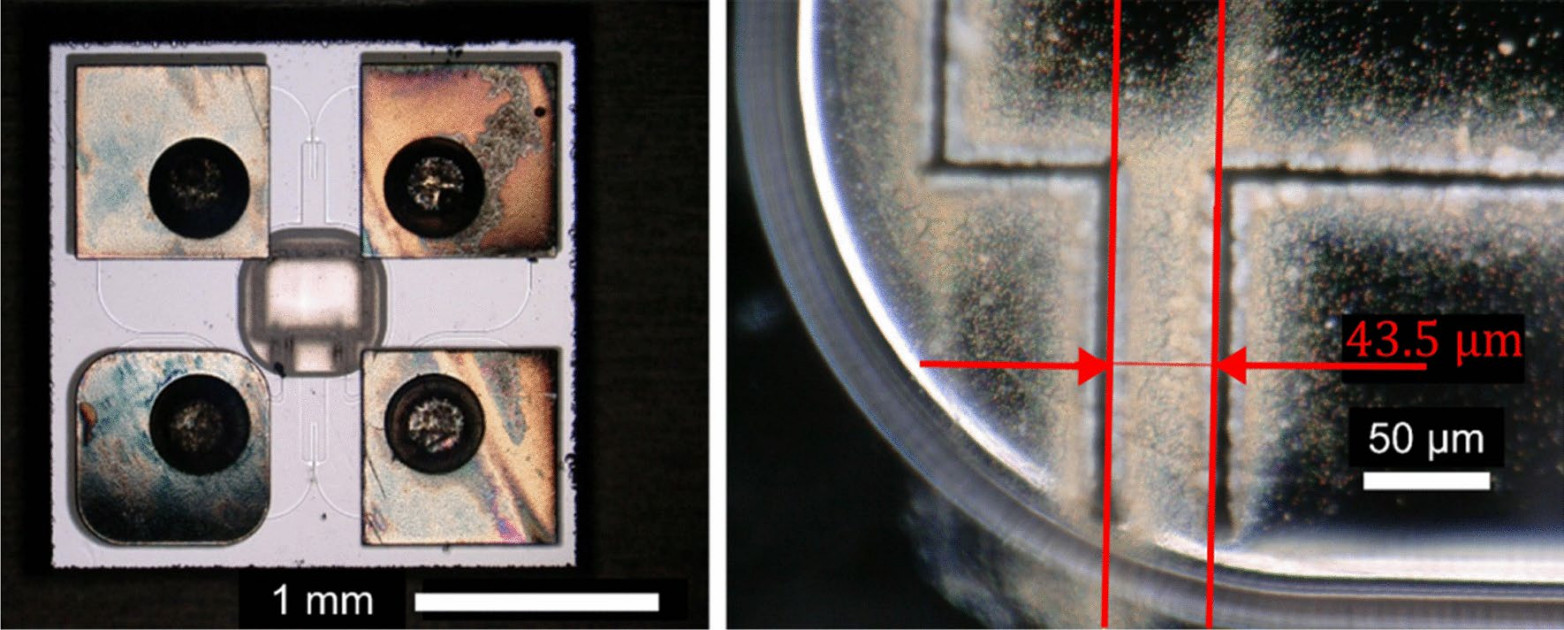
: left: Bottom-view micrograph depicting the silicon resonator, the doping scheme (cf. also Figure 7), and the leadless chip-scale package. Right: Detailed top-view micrograph illustrating the deviation of the laser structured left slender rod width (43.5 µm) from the nominal design width (50 µm), and the limited precision of laser-cutting.

**Fig. 7:**
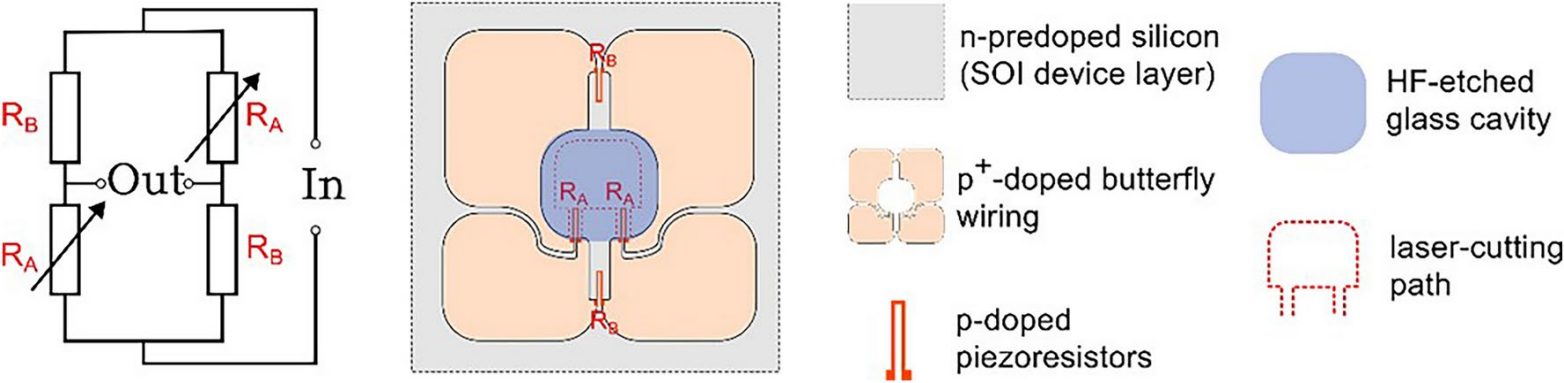
Schematic illustration of the MEMS vibrometer, exposing functional elements and basic geometries.

The MEMS vibrometer was manufactured as described in previous works ^15,22^. Only the p and p+ doping schemes and the laser-cutting path were adjusted to form a structure suspended by two slender rods. Due to the laser’s Gaussian beam profile and limited scanning precision, the silicon structures differ from the target shapes as shown in Figure 6. The cantilever deformation is sensed by p-doped piezoresistive tracks located in the two slender rods (cf. Figure 10). These piezoresistive tracks are wired together with reference piezoresistive paths located on the bulk frame to form a Wheatstone circuit with two strain-dependent resistors R_A_ and two strain-independent complementary resistors R_B_. Figure 7 illustrates the adjusted electromechanical structure of the sensor.

### Methodology of the numerical modal analysis of the resonator

SOLIDWORKS 2022 (Dassault Systèmes SOLIDWORKS Corp.) software was used to create a 3D model. A mesh with dynamic size is created for the resonator as sketched in Figure 5. The material parameters were assigned to represent monocrystalline silicon in <100> orientation. Details of the meshing and the material settings are given in Table 1.

**Table 1:** Input parameters for the FEM model.

Parameter	Quantity
Number of elements; Nodes	1605; 3362
Element type	Tetrahedral 10-Node Element
Standard element size	30 µm
Young’s Modulus	162.71 MPa
Poisson’s ratio	0.27

For performing modal- and transient analysis in order to prove the required dynamic properties, ANSYS 2022R2 (ANSYS GmbH, Germany) software was used. For the numerical modal analysis, ANSYS’ modal response module was utilized. A fixed boundary condition was applied to the slender rod interface towards the sensor frame. The vibration modes and their corresponding frequencies were determined. The strain in the slender rods at the positions of the piezoresistors was evaluated using ANSYS’ dynamic response module. The sensor frame was excited in out-of-plane direction using a linear sweep from 0 Hz to 600 kHz with a peak-to-peak amplitude of 1 nm. For the numerical transient analysis based on the modal analysis, ANSYS’ transient structural module was utilized. The sensor frame was excited with displacement signals representing GUW bursts. As a result, the transient displacements and strains for any node of the mesh could be obtained.

### Setup for experimental modal analysis

**Fig. 8:**
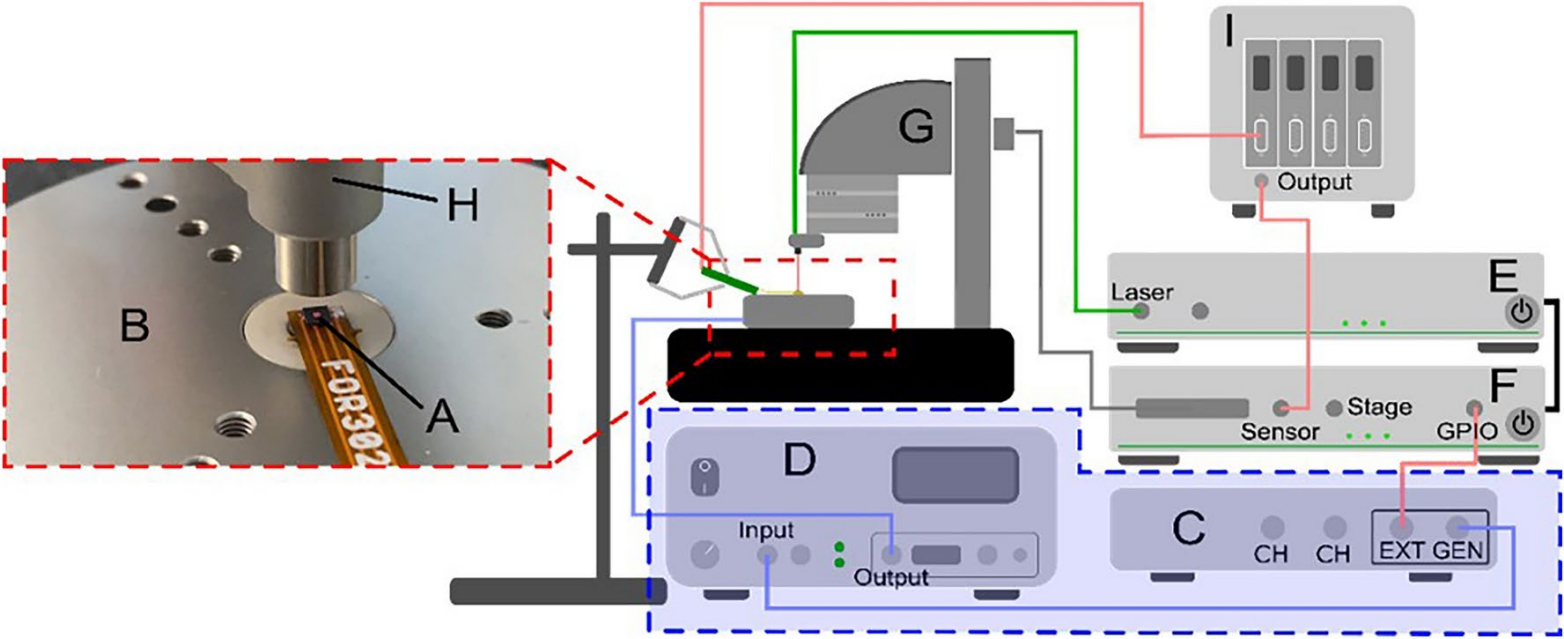
Schematic representation of the experimental setup for modal- and transient analysis. Blue color marks those devices, which were used only for experimental transient analysis.

To confirm the concept of the MEMS vibrometer and to characterize the dynamic response, modal analysis and transient analysis were performed experimentally using a setup as sketched in Fig. 8. The MEMS vibrometer (A) was fixed onto a high-frequency shaker stage (B), driven by a signal generator (C) optionally connected with a piezo amplifier (D) (PD200, PiezoDrive, SI Scientific Instruments GmbH, Gilching, Germany) for transient analysis. The local displacement of the oscillator was measured by an optical vibrometer (E–H) (PicoScale vibrometer by SmarAct GmbH, Oldenburg, Germany), including a laser Michelson interferometer integrated with a confocal microscope, which could be positioned relative to the sample in *x, y, z* – direction. Amplitude and phase of displacements were captured by means of an integrated lock-in amplifier. The sensor’s Wheatstone circuit was driven and evaluated by a signal conditioning module (I) (DAQP-BRIDGE-B, DEWETRON GmbH, is, Austria).

For experimental modal analysis, the amplitude spectrum at Point 2 in Fig. 10 was captured and resonances were identified. In a next step, mode shapes were captured by scanning the cantilever structure pixel by pixel during excitation in resonance. From this data, the modal shapes were reconstructed. For experimental transient sensor characterization, the signal for the shaker stage was amplified using the piezo amplifier and GUW-like bursts were used for excitation. The transient displacements at Points 1 and 2 in Fig. 10 were captured while at the same time the electrical sensor output was recorded.

## Results

### Modal analysis

**Fig. 9:**
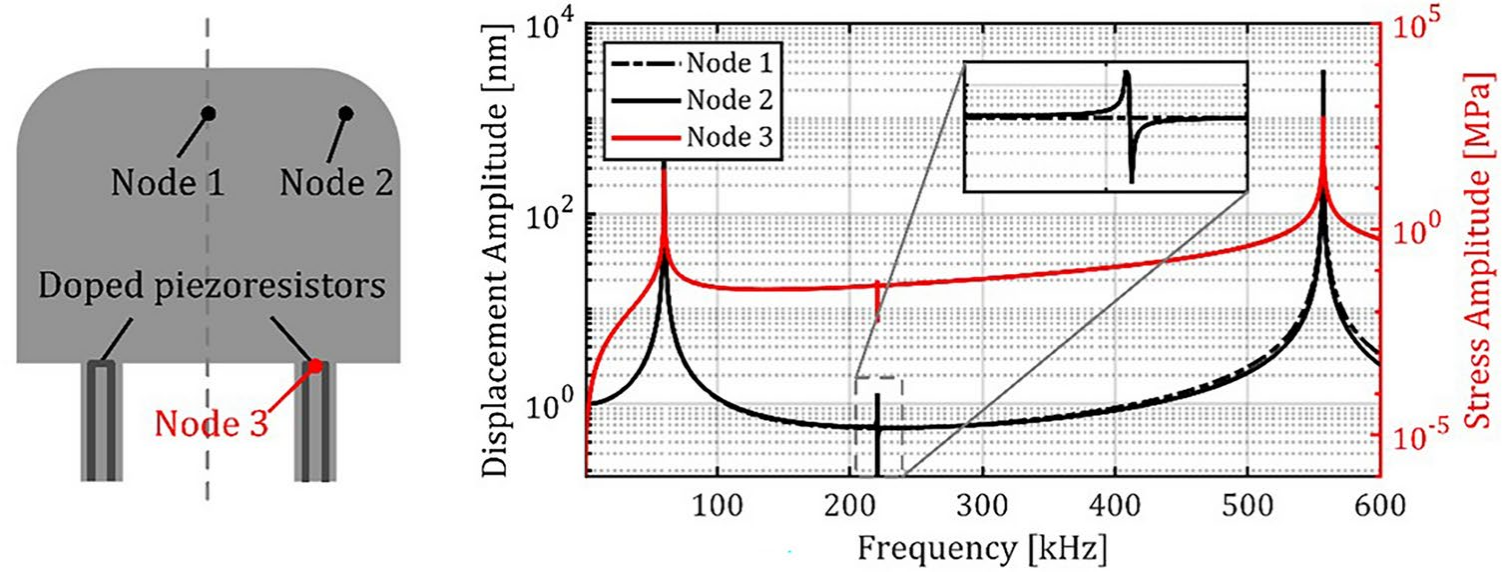
Numerically obtained displacement and stress frequency responses of the slender rod oscillator for three positions (Node 1 to 3).

As the stimulus spectrum is near 100 kHz, the considered frequency range for the modal analysis spans from 0 Hz to 600 kHz including only the first three resonator modes. Figure 9 illustrates the numerically obtained displacement frequency responses at certain positions (Nodes 1 to 3). Node 1 was chosen to exclusively identify bending modes. Node 2 was chosen to identify all of the first three modes. Node 3 was chosen to obtain the normal stress amplitudes (y-axis, cf. Figure 5) which qualitatively represent the piezoresistive response in one of the slender rods. For these positions, two clear resonance peaks were observed at the frequencies 60 kHz and 567 kHz which represent the cantilevers first and second bending mode. At Node 2 and Node 3 a small bipolar peak was observed at 225 kHz, representing the first torsional mode but as expected not at Node 1.

**Fig. 10:**
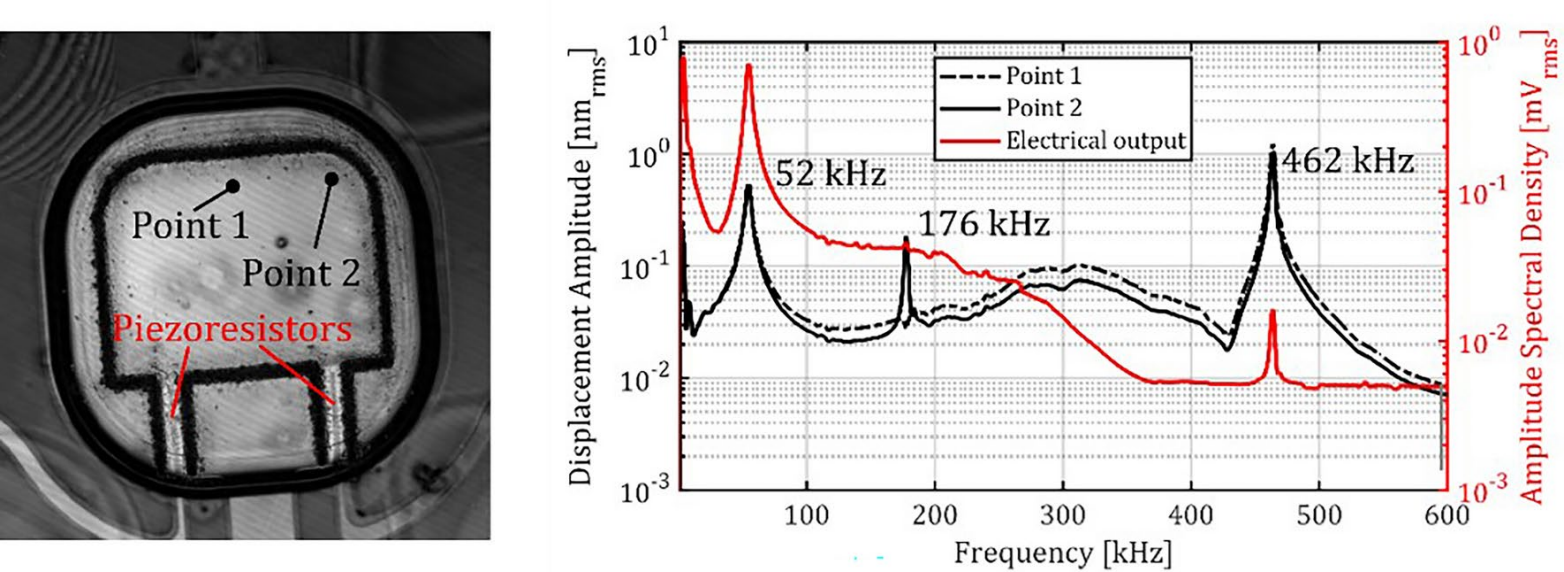
Spectra of displacements optically measured at Point 1 and Point 2 as well as of amplified output signals of the Wheatstone circuit built with piezoresistors wired as sketched in Fig. 7.

Figure 10 shows the displacement spectra experimentally obtained for an exemplary sensor at positions Point 1 and Point 2 together with the spectrum of the sensor’s electrical output. In all these spectra, peaks could be observed at 52 kHz and 462 kHz, representing the resonator’s first and second bending mode resonances. Only in the spectrum obtained at Point 2, an additional peak at 176 kHz is observed, which represents the first torsional mode. The torsional mode cannot be observed at Point 1 (for the same reason as at Node 1 in the numerical analysis) and not in the electrical sensor signal, since it is suppressed by electrical differential mode rejection in the Wheatstone bridge. For frequencies > 200 kHz, the sensor output decreases due to the signal conditioning module, which attenuates signals by –20 dB per decade for frequencies > 200 kHz. Although parasitic contributions from capacitances that form between doped areas (see Fig. 7) can in principle also lead to an attenuation at higher frequencies, a second cut-off frequency cannot be recognized in the output spectrum of the signal.

**Fig. 11:**
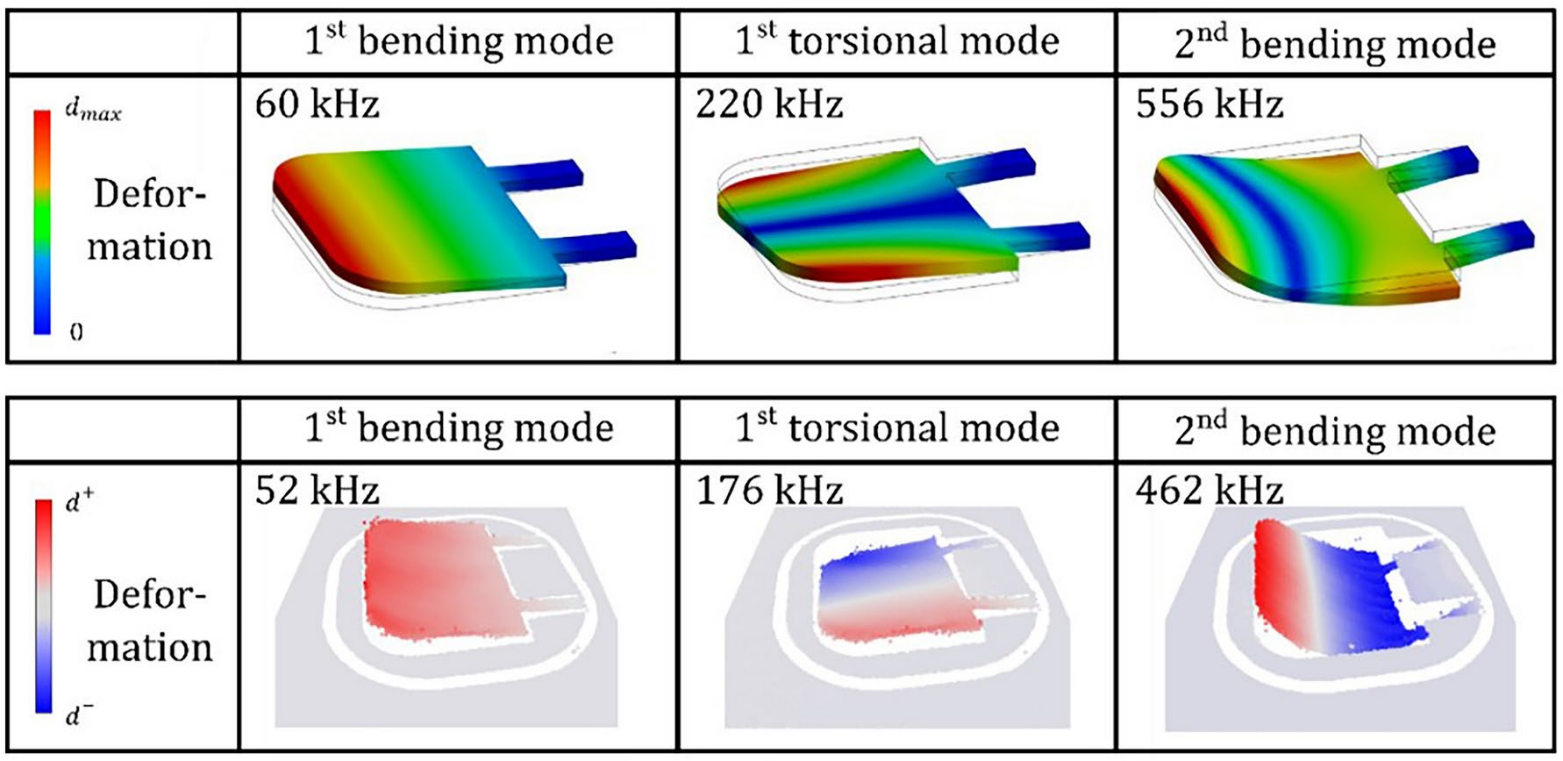
The first three vibration modes obtained in numerical modal analysis (top) and in experimental modal analysis (bottom).

Small deviations between the experimentally and numerically obtained resonance frequency values are due to manufacturing tolerances (as discussed in Sect.  “Materials and methods”), damping caused by viscous friction as well as an inaccurate representation of boundary conditions, stiffnesses and the excitation in the simulation. Figure 11 shows numerically obtained deformation patterns as for the first three resonances i.e., the first bending mode, the first torsional mode and the second bending mode together with the respective resonance frequencies. Furthermore, the corresponding experimentally observed flexible body modes are given, which are in good agreement with the numerically obtained ones and are found at resonance frequencies that deviate only slightly.

### Transient analysis

**Fig. 12:**
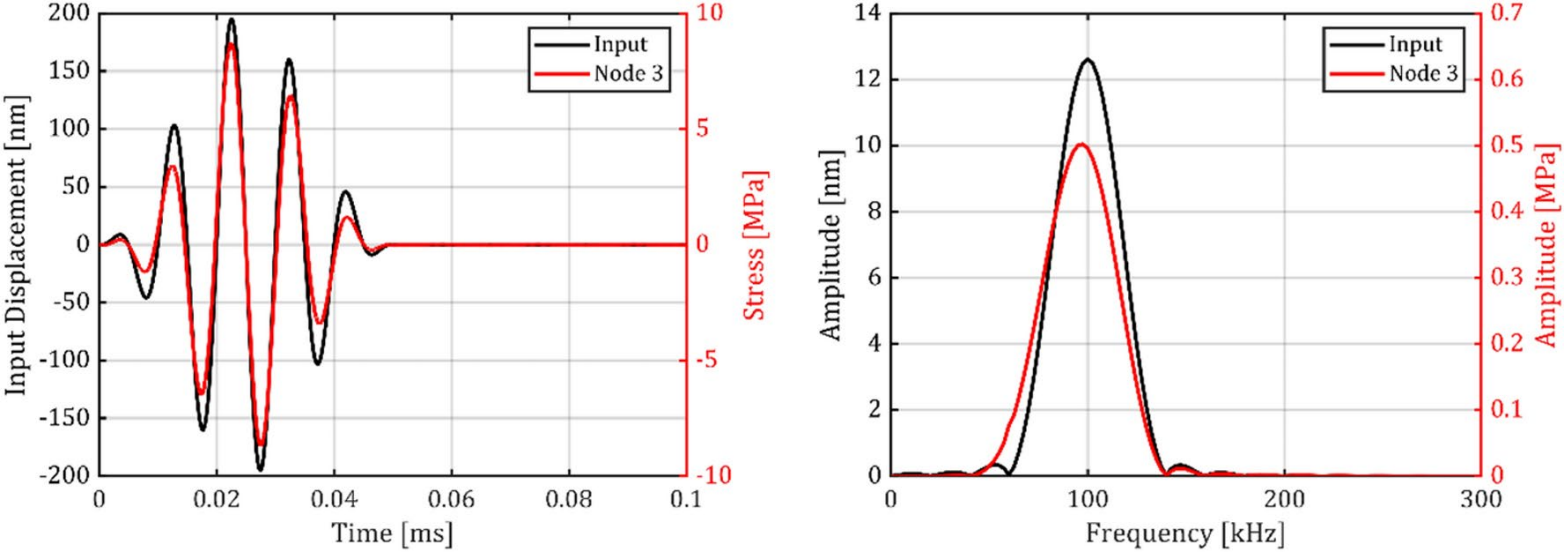
Results of the numerical (FEM) transient analysis in time domain (left) and frequency domain (right) as obtained with a burst input signal and the displacement reaction at Node 3.

Figure 12 presents the results of numerical transient analysis, in time and frequency domain presentation. It shows that the numerical Hanning-window burst excitation signal with a center frequency of ${\omega }^{*}/2\pi =100\,\text{kHz}$ and a window length of $2 \pi / \omega = 50 \;\mu s$ causes a transient stress signal at Node 3 with good correlation to the excitation signal. In the center of the burst, the signals are perfectly in phase. However, at the first half of the burst, a slight negative, and in the second half, a slight positive deviation in period duration can be recognized. In the frequency domain, a shift of − 3 kHz of the central frequency can be recognized between the Node 3 stress and the input displacement.

**Fig. 13:**
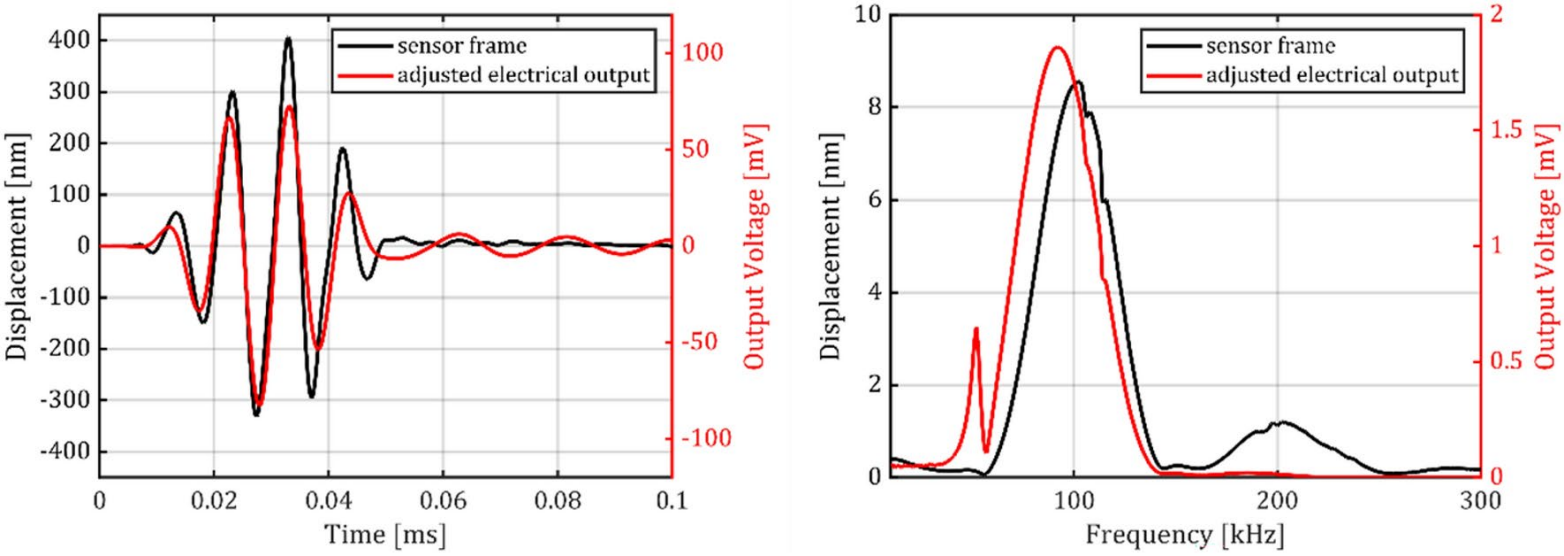
Displacement of the sensor frame as input and the MEMS vibrometer setup output voltage by experimental transient analysis.

Figure 13 presents the results of the experimental transient analysis in the time and frequency domain, where an 85° phase shift due to the lowpass character of the utilized Wheatstone amplifier was compensated by shifting the electrical output by − 2.36 µs for better visual comparability. It shows that the electrical Hanning-window burst excitation signal with a center frequency of ${{\omega }^{*}/2\pi =100\,\text{kHz}}$ and a window length of ${2\pi /\omega = 50 \;\mu s}$ was converted into an optically measured displacement signal (laser spot located at the sensor frame). The frequency domain displacement plot of the sensor frame confirms that the resonator was exposed to a GUW-like motion with only minor deviations and a center frequency of 100 kHz, but also to a second harmonic which showed up at $200\,\text{kHz}$. The MEMS vibrometer’s signal (amplified Wheatstone bridge output signal) correlated with the displacement stimulus even if not as direct as in the simulations. The electrical signal is shifted in phase and frequency (as expected from the numerical analysis) and also shows some oscillation continuing after the burst at lower frequency. The sensor signal exhibited a center frequency of 92 kHz and also responded with a resonance peak at 52 kHz, representing the oscillator’s first bending mode but did practically not respond to the excitation at $200\,\text{kHz}$.

## Discussion

### Modal analysis and electrical mode rejection

**Fig. 14:**
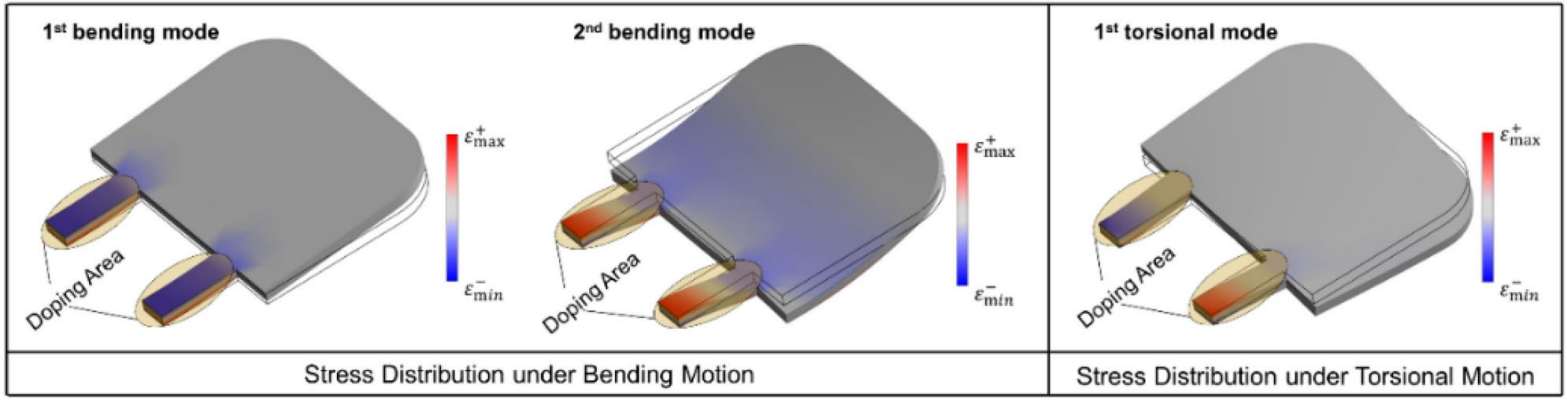
Schematic stress distribution of the oscillator at bending and torsional modes.

The results confirm that the presented MEMS vibrometer is suitable to transduce the displacements induced by passing GUW into an electrical signal as could be predicted in the numerical analysis. The presented oscillator structure had its harmonic mode frequencies sufficiently separated from the GUW band and thus the oscillator had only minimal resonant coupling with the GUW at a center frequency of 100 kHz. Due to the character of GUW typically being narrow band bursts, the resonator can always be designed to show an approximately constant transfer amplitude within the bandwidth of the GUW even if the excitation center frequency would be varied. The torsional mode appeared less strong in the simulations than in the experiments. This is because the excitation is idealized in the simulation along only one axis but in the experiments, the shaker stage potentially generates more complex vibrations which can more strongly excite the torsional mode. For the presented oscillator design with a two slender rod suspension, the first torsional mode frequency is located between the first and second bending mode frequencies. As illustrated in Fig. 14 symmetrically placed slender rods carrying the piezoresistive tracks opposing strains result when this mode is excited. Signal contributions originating from the torsional motion are therefore suppressed by the electrical differential mode rejection of the Wheatstone circuit.

In the experiments the excitation has an undesired secondary contribution of 200 kHz which is most likely attributed to a second order harmonic of the excitation setup. Although this stage oscillation occurs near the oscillator’s torsional mode frequency, the sensor signal did not contain a significant component in this spectrum.

### Transient analysis

**Fig. 15:**
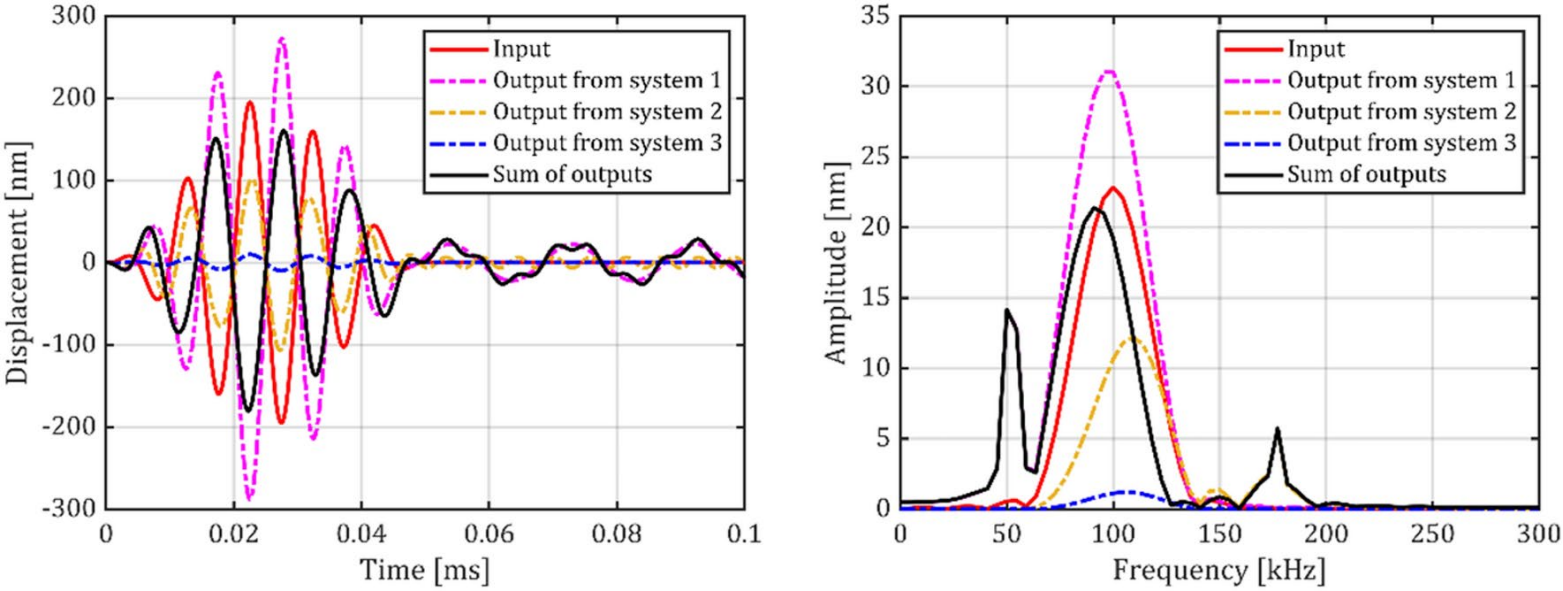
Analytically derived responses of the parallel multimodal oscillator system and its individual resonators to an GUW-like Hanning burst input function. The amplitudes of all resonators were set to the same value.

In order to correctly transduce GUW induced sensor displacements with the MEMS vibrometer, the electrical signal must show a linear response to short ultrasonic displacement bursts. As presented in Sect. “Vibrometer as transducer for GUW bursts”, this is virtually the case, if the stimulus is located in the quasi-free band of the lowest oscillator resonance, and the second oscillator band has near-zero contribution to the output. The output signals (in simulations and experiments) however, showed a slight frequency shift towards lower frequencies, in other words a pseudo-nonlinear response. This phenomenon was more pronounced in the experimental transient analysis; however, the analytical approach is best suited to visualize its cause: In contrast to a situation with sufficient frequency separation between the stimulus and the sensor resonances, as modelled in Sect.  “Vibrometer as transducer for GUW bursts”, the two resonances are close to the center frequency of the GUW-like stimulus. Therefore, the output signal is composed of the outputs of multiple oscillators, as presented in Fig. 15.

**Fig. 16:**
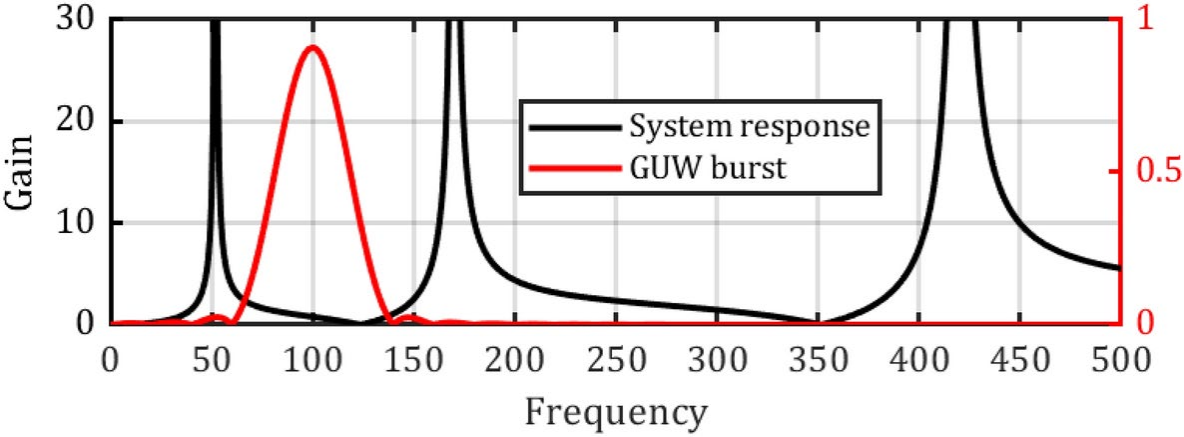
Analytically equivalent model of the mechanical oscillator system $\omega_{1} \approx 0.5 \cdot \omega^{2} ;\omega_{2} \approx 1.7 \cdot \omega^{*} ).$ Presented alongside the excitation spectrum of a 5-cycle Hanning burst with center frequency ${\omega }^{*}/2\pi = 100\,\text{kHz}.$

Each single mode resonator (1–3) transduces the input signal according to its individual transfer function. Resonator 1 responds with a − 180° phase shift due to excitation in its quasi-free band, resonators 2 and 3 are excited in their quasi-static bands and therefore respond with approx. 0° phase shifts. Particularly noteworthy is that each individual resonator frequency, as well as the resulting system output shows the pseudo- nonlinear behavior with a shift in center frequency. With a slope in the amplitude response functions (cf. Figure 3), each resonator contributes differently to the higher and lower frequency components of the burst excitation. The output spectrum transforms towards higher frequencies for quasi-statically excited resonators, and towards lower frequencies for resonators in quasi-free excitation. The same applies for the response function of the combined system, given in Fig. 16.

The sum of the single mode resonator outputs represents the multi modal sensor output. Based on the superposition model the multimodal sensor response is the sum of the single mode transfer functions. This leads to a destructive interference in the frequency range of the excitation spectrum, which expresses itself as a local mainly negative slope of the combined system’s amplitude response function. Consequently, the center frequency of the system’s output appears shifted towards lower frequencies. If, however, the parasitic mode’s contribution to the overall sensor signal is eliminated due to e. g. the presented electrical differential mode rejection, the systems transfer behavior can be effectively improved.

## Conclusion and outlook

Inertial MEMS vibrometers can transduce GUW-burst induced displacements into electrical signals. Their complex multi modal transfer behavior, however, requires prior knowledge of the input signal in terms of frequency and bandwidth, so that resonator modes can be adjusted to provide sufficient response in the quasi-free band of one mode. Even for an optimally adjusted resonator, the parasitic contributions of other modes must also be considered. Mode adjustments by micromechanical design can be realized and evaluated by means of FEM and experimental modal- and transient analysis methods Nevertheless, the pseudo-nonlinearity observed from the investigated sensor can be considered as not critical for the structural health monitoring application. In future work, it has to be investigated, whether the sensitivity must be improved to record ultrasonic waves guided over longer distances that occur in relevant applications, where displacements are expected to be at least one order of magnitude smaller than on the shaker stage.

**Fig. 17:**
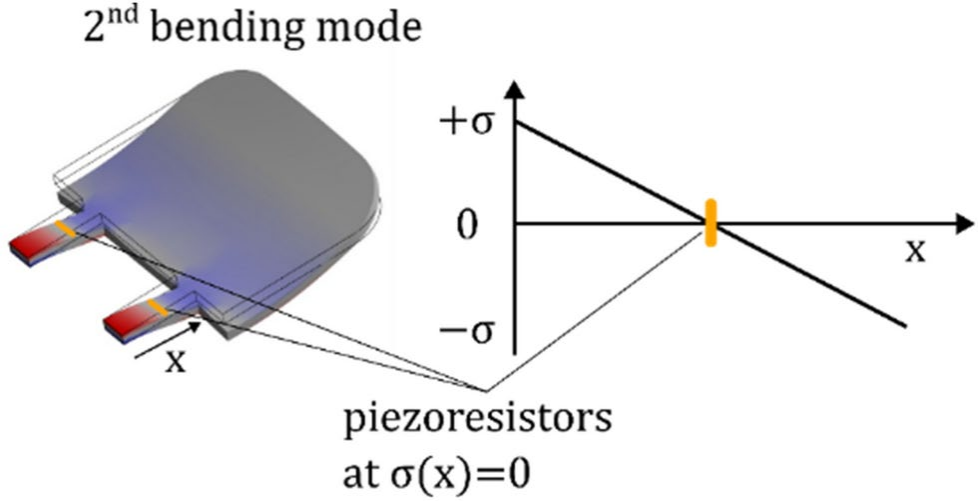
Concept for a doping scheme which suppresses the undesired contribution from first torsional and second bending mode to the sensor signal. The distribution of stress $\upsigma (x)$ within the slender rods indicates a position with $\sigma =0$.

Due to the ability of the electrical differential-mode rejection to effectively suppress the parasitic contribution from the first torsional mode in the sensor signal, a further mode rejection approach shall be investigated in future work: Piezoresistors, locally doped into the location of the second bending mode’s stress-node will be insensitive to second bending mode deformations as sketched in Fig. 17. The aim is to provide an almost undistorted displacement sensitive operational band at frequencies above the first resonance frequency, and to thereby minimize the pseudo-nonlinear response to burst signals.

## Data Availability

The datasets used and/or analyzed during the current study are available from the corresponding author on reasonable request.
